# A phenomenological density-scaling approach to lamellipodial actin dynamics^†^

**DOI:** 10.1098/rsfs.2014.0006

**Published:** 2014-12-06

**Authors:** Alexandre Lewalle, Marco Fritzsche, Kerry Wilson, Richard Thorogate, Tom Duke, Guillaume Charras

**Affiliations:** 1 London Centre for Nanotechnology, University College London, 17–19 Gordon Street, London WC1H 0AH, UK; 2 Department of Cell and Developmental Biology, University College London, Gower Street, London WC1E 6BT, UK; 3 Institute for the Physics of Living Systems, University College London, Gower Street, London WC1E 6BT, UK

**Keywords:** lamellipodium, actin network, photoactivation, actin dynamics

## Abstract

The integration of protein function studied *in vitro* in a dynamic system like the cell lamellipodium remains a significant challenge. One reason is the apparent contradictory effect that perturbations of some proteins can have on the overall lamellipodium dynamics, depending on exact conditions. Theoretical modelling offers one approach for understanding the balance between the mechanisms that drive and regulate actin network growth and decay. Most models use a ‘bottom-up’ approach, involving explicitly assembling biochemical components to simulate observable behaviour. Their correctness therefore relies on both the accurate characterization of all the components and the completeness of the relevant processes involved. To avoid potential pitfalls due to this uncertainty, we used an alternative ‘top-down’ approach, in which measurable features of lamellipodium behaviour, here observed in two different cell types (HL60 and B16-F1), directly inform the development of a simple phenomenological model of lamellipodium dynamics. We show that the kinetics of F-actin association and dissociation scales with the local F-actin density, with no explicit location dependence. This justifies the use of a simplified kinetic model of lamellipodium dynamics that yields predictions testable by pharmacological or genetic intervention. A length-scale parameter (the lamellipodium width) emerges from this analysis as an experimentally accessible probe of network regulatory processes.

## 1. Introduction

The dynamics and regulation of actin-filament growth and decay play a central role in the generation of force for protrusion [1,2]. During migration, cells become polarized and form a thin flat veil-like protrusion called a lamellipodium at the cell front. Similar protrusions also form during cell spreading on a surface, sometimes extending around the whole cell periphery [3]. In both cases, at the molecular level, forward-directed growth of an actin filament network tethered to the substrate is thought to provide the motive force pushing the cell membrane forward via a ratchet-like mechanism that rectifies thermal fluctuations in the filament–membrane gap [1,2,4–6]. The strength of interfacing of the actin network to the substrate determines what portion of new polymerization contributes to forward protrusion [7]. If filaments are tightly tethered to the substrate, polymerization generates forward protrusion but in the absence of coupling, the actin network slips over the substrate, giving rise to an immobile leading edge and retrograde actin flow. Non-motile cells display a constant retrograde flow of F-actin, whereas motile cells may show a combination of forward protrusion and retrograde flow [8,9].

Because of the physiological importance of lamellipodial protrusion and migration in development, immunity and cancer, the molecular mechanisms underlying actin dynamics in the lamellipodium have intensely been studied. The current consensus is that activation of the Arp2/3 complex at the leading membrane by WAVE family proteins leads to the formation of a branched network of actin filaments with their fast growing barbed-ends pointing towards the membrane and providing the motive force for protrusion [10–13]. Filament growth against the front membrane displaces the existent F-actin network rearwards [12,14,15]. Away from the front membrane, filament growth is progressively blocked by association of capping proteins to filament barbed-ends and depolymerization occurs through a combination of monomer loss from the pointed-ends and severing. A growing number of proteins participating in actin network dynamics have been identified with apparently redundant functions. For example, barbed-ends can be capped either by heterodimeric capping-protein or Aip1, with each binding preferentially to subsets of filaments at different stages of adenosine triphosphate (ATP) hydrolysis [16,17]. Severing and end depolymerization appear to result primarily from the action of actin depolymerizing factor (ADF)/cofilin though the relative importance of end depolymerization versus severing remains a matter of debate [18]. To add further complexity to this picture, the actin network topology can be modified through the action of a number of proteins (such as myosin, coronin, cortactin), which can also affect actin turnover dynamics [19]. Though *in vitro* study of individual proteins has proven a powerful method for understanding function, it is becoming increasingly apparent that protein function depends strongly on environmental conditions. For example, cofilin family proteins generally lead to net depolymerization of the actin network [20–22], but under some conditions the creation of new barbed-ends associated with severing has been shown to lead to net polymerization and lamellipodial extension [23]. This complexity presents a significant challenge to understanding the global behaviour of the lamellipodium and its function at the cell level.

Despite the wealth of experimental studies, the spatial organization of actin turnover within the lamellipodium also remains a matter of debate; yet, it underlies the very basis of structural integrity of the lamellipodium and its physiological function. It is generally argued that the leading-edge boundary acts as the main source of F-actin while the lamellipodium body behaves as a sink [15]. Although incorporation is highest at the tip of the leading edge owing to its high barbed-end density, a point of controversy has been whether incorporation occurs exclusively there [14,24–26] or over a more extended region [15,27]. Evidence for the former stems in particular from photobleaching experiments that show a ‘recovery front’ of actin fluorescence that sweeps from tip of the leading edge through the lamellipodium at the speed of retrograde flow [14] (‘convective recovery’), whereas evidence for the latter comes from the detection of speckles of actin monomer incorporation throughout the lamellipodium (‘reactive recovery’) [15,16,27].

Numerical modelling studies have explored the complex interplay between protein activity, network structure and protrusion to understand actin dynamics on a global level, but to date, most models have relied on explicit modelling of biochemical reactions and network structure (see reviews in [28,29]). Though informative, such approaches have limitations. Indeed, the *in vitro* reaction rate measurements they are based on may not reflect *in vivo* rates and, despite a growing number of known pair-wise protein interactions, our knowledge is far from complete. Finally, the vast number of reactions and proteins involved can obscure the search for emergent properties of lamellipodial actin dynamics. In summary, despite a wealth of experimental and theoretical work as well as a growing consensus on the biochemical ingredients at play in lamellipodial actin turnover [13], fundamental questions remain controversial, including (i) the location of monomer insertion in the lamellipodium, (ii) the turnover of actin in the bulk of the lamellipodium, (iii) what controls actin density, and (iv) what parameters should be measured to characterize the impact of perturbations.

Here, we examined these questions in two cell types with well-developed lamellipodia but very different dynamics: HL60 neutrophil-like cells that are highly motile and show strong coupling between their lamellipodial actin and the substrate; and B16-F1 mouse melanoma cells, with a stationary lamellipodium and high retrograde flow. We measured the spatio-temporal dependence of actin incorporation and dissociation kinetics using cells stably expressing actin monomers doubly labelled with mRFP and photoactivatable GFP (PA-GFP), and explored the commonalities between both cell types to derive a general empirical model for lamellipodial actin turnover dynamics. Our analytical approach was designed to formalize and explain global features of the lamellipodial actin network with minimal assumptions on its detailed structure or biochemical behaviour. We find that the characteristic width of the lamellipodium, a directly measurable parameter, emerges as a phenomenological handle to assess the impact of chemical or genetic interventions.

## 2. General experimental approach

We sought to characterize general properties of steady-state lamellipodial actin turnover dynamics by examining the commonalities between two cell types with prominent lamellipodia: HL60 neutrophil-like cells, which are highly motile, and B16-F1 cells, which are stationary. Both cell types expressed actin monomers simultaneously tagged with two fluorophores: mRFP and a PA-GFP that only fluoresces once activated with 405 nm light [22,30]. These two fluorophores provide experimental degrees of freedom to discern actin growth and decay kinetics in the steady state phenomenologically. For all cells, steady-state behaviour was identified by confirming the absence of notable change in the overall F-actin density distribution (measured with the mRFP fluorescence intensity) and in the overall cell shape over several minutes, several-fold longer than the typical actin turnover time (approx. 1 min for both HL60 and B16-F1; see the electronic supplementary material, §C.2).

We inferred the turnover dynamics by selectively photoactivating regions of a cell and measuring the intensity decay of both fluorophores simultaneously. At any given location at times long compared to the characteristic time associated with actin monomer diffusion, fluorescence intensity may vary due to (i) actin monomer dissociation kinetics (‘reactive dynamics’) and (ii) retrograde flow of the actin network (‘convective dynamics’) when the actin network is not stationary relative to the substrate. Therefore, to differentiate between these contributions in each cell type, we first established the presence or absence of retrograde flow by photoactivating the PA-GFP-actin in a small region of the lamellipodium and then monitoring the PA-GFP fluorescence over time. The mRFP-actin is unaffected by the photoactivating light. Knowledge of the network flow allows the subtraction of the convective component from the total measured fluorescence intensity, and hence the calculation of the (reactive) monomer binding/unbinding kinetics. We define *ω*_+_ and *ω*_−_ as, respectively, the instantaneous phenomenological rates of actin association and dissociation per unit density of F-actin. These rates are purely empirical and are measurable unambiguously and independently of either the underlying biochemical mechanisms or the filament network topology.

**Figure 1.**
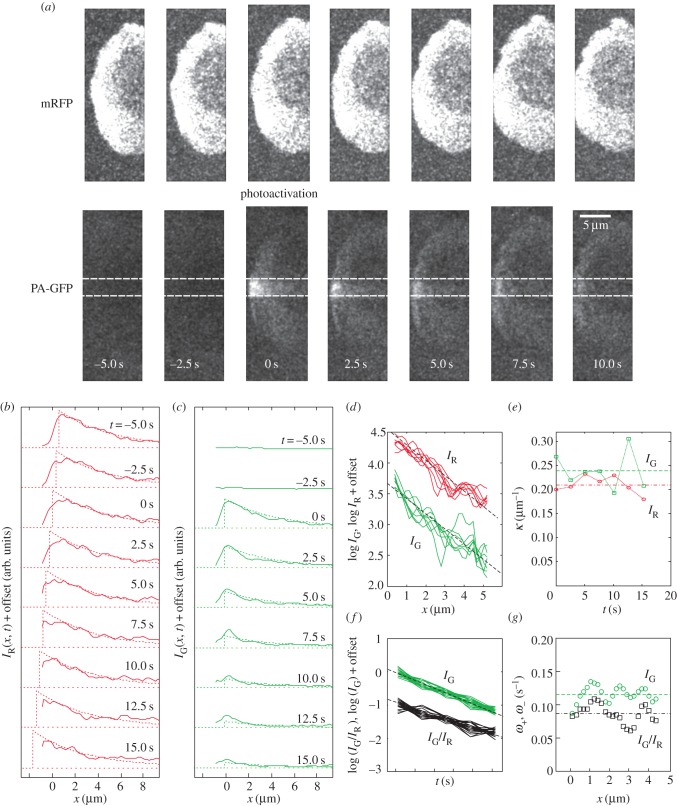
Actin turnover in the lamellipodium of motile HL60 cells. (*a*) Photoactivation of PA-GFP actin monomers at time *t* = 0 of a rectangular strip (contained within the dashed white lines) across the lamellipodium of an HL60 cell. The mRFP channel, measured simultaneously, is unaffected by the photoactivation. (*b*) mRFP intensities *I*_R_(*x*, *t*), averaged across the strip, at time intervals of 2.5 s, together with fits to a travelling exponential function over a constant background level (dotted curves; see §4). The front of the distribution was determined from the intensity maximum, which progresses leftward with velocity 0.11 ± 0.01 μm s^−1^. The cell has moved out of the image frame at times 7.5 and 10.0 s, and the corresponding distribution fronts were therefore estimated by extrapolation. (*c*) PA-GFP intensities *I*_G_(*x*, *t*) for the same region and same times as in (*b*). Intensity is zero before photoactivation (*t* < 0). (*d*) Spatial decay: log[*I*_R_(*x*, *t*)] (red lines) and log[*I*_G_(*x*, *t*)] (green lines) obtained from (*b*,*c*) are normalized to their values at *x* = 0 and plotted as functions of *x* for each *t* value. (*e*) The spatial decay constants *κ*, obtained from the slopes of the plots in (*d*), show no notable time dependence. Average values: *κ*_R_ = 0.21 ± 0.01 µm*^−^*^1^ (circles, dotted-dashed line), *κ*_G_ = 0.23 ± 0.03 µm*^−^*^1^ (squares, dashed line) (*n* = 7 points). (*f*) Temporal decay: log(*I*_G_) and log(*I*_G_/*I*_R_) normalized to their values at *t* = 0 and plotted as functions of time for each *x*. (*g*) Temporal decay constants *ω*_−_ (circles) and *ω*_+_ (squares) were derived from the slopes in (*f*) (equations (2.2) and (2.3)) and show negligible *x* dependence. Average values: 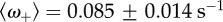 (dashed line), 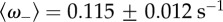 (dotted-dashed line) (*n* = 24 points). (Online version in colour.)

**Figure 2.**
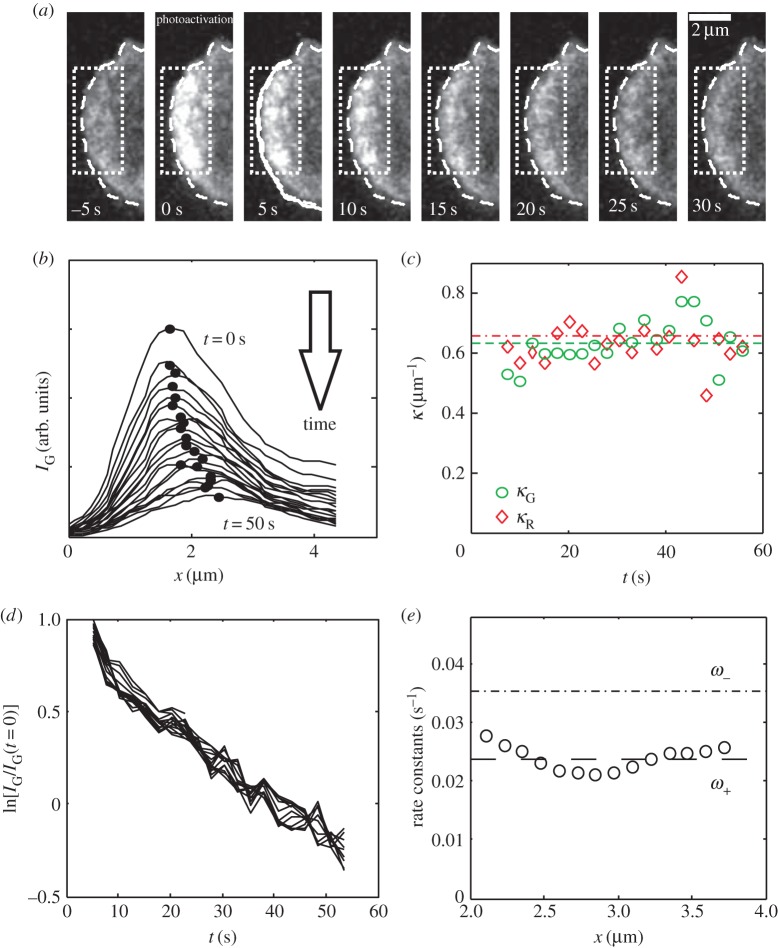
Actin turnover in the lamellipodium of stationary B16-F1 cells. (*a*) Photoactivation at time *t* = 0 of a rectangular region (white dotted lines) in the lamellipodium of a B16-F1 cell. (*b*) PA-GFP intensity *I*_G_(*x*, *t*) after photoactivation plotted as a function of *x*, at 2.5 s time intervals. The intensity maximum *x*_max_ retreats at constant velocity *v* = 1.0 µm min^−1^ (see also the electronic supplementary material, figure D.1*d*,*f*). (*c*) The spatial decay constants *κ*_G_ of the exponential regime of the PA-GFP intensity distribution *I*_G_(*x*, *t*) (green circles, mean value 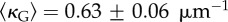, *n* = 20 points, dashed line) and *κ*_R_ of the mRFP distribution *I*_R_(*x*, *t*) (red diamonds, 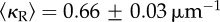, *n* = 20 points, dashed-dotted line), plotted as functions of time. (*d*) log[*I*_G_(*x*, *t*)] plotted as functions of *t* for each *x* > *x*_max_. (*e*) The temporal decay constants *ω*_−_ (circles, mean value 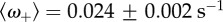, *n* = 14 points, equation 2.5)), obtained from the slopes in (*d*) plotted as a function of *x*. The dashed-dotted line indicates *ω*_−_, derived as *ω*_+_ + *κv* ≈ 0.035 ± 0.003 s*^−^*^1^ (equation 2.4)). (Online version in colour.)

In HL60 cells, the spatio-temporal dependence of *ω*_+_ and *ω*_−_ was measured by photoactivating a small rectangular region spanning the length of the lamellipodium. In all the cells analysed, photoactivation altered only the PA-GFP fluorescence but not the mRFP distribution, suggesting that it had no effect on the regulatory kinetics of actin turnover [31] (figures 1 and 2; electronic supplementary material, D.1, D.2). In the network rest frame of HL60 cells, which show no retrograde flow, the mRFP fluorescence intensity at a location *x* and at a time *t*, *I*_R_(*x*; *t*), is proportional to the total local F-actin density and any change therefore reflects the net balance of growth (*ω*_+_) and decay (*ω*_−_):2.1
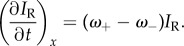
On the other hand, provided that the photoactivated region is small compared with the cell volume, reincorporation of photoactivated monomers after dissociation occurs with a negligible probability and therefore the temporal evolution of the PA-GFP intensity *I*_G_(*x*; *t*) predominantly reflects decay kinetics only. The size of the photoactivated region was sufficiently small to minimize reincorporation of photoactivated monomers but sufficiently large for our measurements to possess a good signal-to-noise ratio. We expect reincorporation to be most significant immediately after photoactivation because the proportion of fluorescent to non-fluorescent monomers is greatest [32,33], and therefore our data fitting was done over the largest possible time spans. Thus, in the absence of retrograde flow, *ω*_+_ and *ω*_−_ are determined from the decays of the distributions *I*_R_ and *I*_G_/*I*_R_:2.2
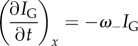
and2.3
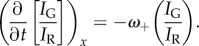
Provided *ω*_+_ and *ω*_−_ are time- and location-independent (and as confirmed below), both *I*_R_ and *I*_G_ have an exponential solution *I*_R,G_ ∝ exp(*κ*x), where2.4
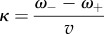
defines a characteristic length scale and *v* is the speed of the cell motion. Details of the analysis are given in the electronic supplementary material, §A.

B16-F1 cells are stationary but display a constant retrograde flow (see Results). The network dynamics is formally identical to the HL60 cells, albeit after a change of reference frame *x* → *x* − *vt* (see §3.1). Thus, equation (2.2) remains true in the network rest frame. However, in the substrate rest frame, the combined convective and reactive contributions to fluorescence change give2.5
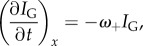
which allows the determination of *ω*_+_ from the PA-GFP distribution *I*_G_ (electronic supplementary material, §A). Equation (2.4) then yields *ω*_−_ = *ω*_+_ + *κv*.

## 3. Experimental results

### 3.1. Network flow

First, we determined the actin network flow dynamics in HL60 cells travelling under agar, a condition in which they can navigate chemoattractant gradients but possess broader lamellipodia (5–10 µm width) than on two-dimensional substrates [34]. After verifying that the observed cell was at steady state (see the electronic supplementary material, §C.2), we photoactivated small circular regions (diameter < 1 µm) and monitored their displacement while the cell moved steadily. The photoactivated spots remained stationary relative to the substrate while the leading edge membrane advanced at a constant velocity (*v* = 0.086 ± 0.018 µm s^−1^, 15/15 cells; electronic supplementary material, videos S1, S2). There was therefore no retrograde flow (i.e. no ‘convective’ contributions to recovery) in the HL60 cells and variations in fluorescence at a given substrate location resulted exclusively from the binding or release of actin monomers (‘reactive’ fluorescence recovery).

B16-F1 cells possess comparatively narrower lamellipodia (average width 1.2 ± 0.1 μm, *n* = 28 cells) and, in contrast to HL60 cells, their leading edge is essentially stationary. We measured retrograde flow velocity by photoactivating a thin band of the leading edge. The peak in fluorescence moved towards the cell rear at a constant speed, indicating a steady retrograde flow of the F-actin network (*v* = 0.018 ± 0.014 µm s^−1^, 10/10 cells observed; electronic supplementary material, video S3).

These results imply that (i) in both cell types, the F-actin flow away from the leading-edge membrane had a single, constant velocity relative to the front edge, hereafter called *v*, (ii) there was therefore no detectable compaction of the actin network within the lamellipodium, and (iii) different regions of the network did not interpenetrate. The variations in F-actin density must therefore result from actin filament association and dissociation at sites advected along with the network with velocity *v*. These observations allow the reactive dynamics in both cell types to be compared directly after a change of reference frame (*x* → *x* − *vt*).

### 3.2. Actin association and dissociation rates, *ω*_±_

#### 3.2.1. HL60 cells

To determine the empirical rates *ω*_+_ and *ω*_−_ in steady-state HL60 cells, we measured *I*_R_(*x*, *t*) and *I*_G_(*x*, *t*) by photoactivating a thin strip oriented perpendicular to the leading edge (in between the white dashed lines, figure 1*a*) and averaged fluorescence intensities across the width of the strip (approx. 7.0 µm) to enhance the signal-to-noise ratio. *I*_R_(*x*, *t*) displayed a distinct peak, which corresponded to the position of the leading-edge membrane. This peak moved with a constant velocity, consistent with steady-state motion of the cell (*v* = 0.076 ± 0.015 µm s^−1^, figure 1*b*). Plots of log[*I*_R_(*x*, *t*)] revealed an exponential decay with distance from the front (*κ*_R_ = 0.22 ± 0.01 µm *^−^* ^1^, figure 1*d*,*e*; data measured for *x* greater than 1/*κ*_R_ ∼ 5 µm were dominated by noise and were discarded for the analysis). Immediately after photoactivation, the spatial profile of *I*_G_(*x*, *t* = 0) matched *I*_R_(*x*, *t* = 0) because individual monomers were photoactivated with equal probability (figure 1*a*–*c*). Consistent with the absence of retrograde flow (§3.1), the peak in *I*_G_(*x*, *t*) remained stationary while its amplitude decreased with time (figure 1*c*).

To derive experimental measurements of *ω*_+_ and *ω*_−_ at each location *x*, we plotted log[*I*_G_/*I*_R_] and log[*I*_G_], normalized to their values at *t* = 0, as functions of *t* (figure 1*f*), as suggested by equations (2.2)–(2.3). Within experimental error, all the data collapsed onto single straight lines (figure 1*f*), indicating that *I*_G_/*I*_R_ and *I*_G_ decayed exponentially on time scales *ω*_+_ and *ω*_−_ that were constants over most of the lamellipodium width (*ω*_+_ = 0.085 ± 0.014 s*^−^*^1^, *ω*_−_ = 0.115 ± 0.012 s*^−^*^1^, figure 1*g*). In other words, the net rates of F-actin association and dissociation, which are given by *ω*_+_*I*_R_ and *ω*_−_*I*_R_, depend explicitly on the actin density at a given location but not significantly on the distance from the leading edge. This result is confirmed by the fact that the spatial decay constant *κ*_G_ of *I*_G_, obtained from the slope of log[*I*_G_(*x*, *t*)] as a function of *x*, did not vary significantly with time even though its relative position within the leading edge did change (figure 1*d*–*e*). The average value of *κ*_G_ (0.23 ± 0.03 µm*^−^*^1^) was also not significantly different from *κ*_R_ derived from *I*_R_ (*p* = 0.5, figure 1*e*). The persistence of linear behaviour in log[*I*_G_(*x*, *t*)] over the whole *x* and *t* ranges suggests that actin monomers dissociate with uniform probability. The difference *ω*_−_ − *ω*_+_ was always positive and not significantly different from *κv* (*p* = 0.1), as required in the steady state and as predicted by equation (2.4).

Qualitatively similar results were obtained in all cases examined (7/7 cells, figure 8*a*; electronic supplementary material, video S4).

#### 3.2.2. B16-F1 cells

We performed a similar analysis for B16-F1 cells, where strong retrograde flow implies that the actin rest frame is not stationary with respect to the substrate (§3.1; electronic supplementary material, figure D.1). Because the lamellipodia of B16-F1 cells are much narrower than those of HL60 cells, we positioned the activation rectangle over a longer segment of the lamellipodium to allow better averaging (figure 2*a*, white dotted line). Plots of the red fluorescence intensity profiles *I*_R_(*x*, *t*) displayed a prominent peak corresponding to the leading-edge membrane (electronic supplementary material, figure D.1*a*). In contrast to HL60 cells, this peak was stationary (electronic supplementary material, figure D.1*d*), in agreement with lack of movement and steady-state behaviour within the experimental time frame. Immediately after photoactivation, the PA-GFP intensity profile *I*_G_(*x*, *t* = 0) again matched the mRFP profile (figure 2*a*,*b*; electronic supplementary material, figure D.1*b*, top curve). Over time, the amplitude of the peak in *I*_G_(*x*) decreased and its position moved away from the tip of the leading edge with a constant velocity *v* ≈ 1.1 µm min^−1^, consistent with the presence of retrograde flow (electronic supplementary material, figure D.1*b*,*c*).

When we plotted log[*I*_G_(*x*, *t*)/*I*_G_(*x*, *t* = 0)] as a function of *t* at each location *x* on the substrate, all the data collapsed onto the same straight line (figure 2*d*) showing that *I*_G_(*x*, *t*)/*I*_G_(*x*, 0) is an exponential function with a characteristic time scale *ω*_+_ that is both *x*- and *t*-independent in B16-F1 cells (equation (2.5); electronic supplementary material, figure D.1*i*). Results for six different cells are plotted against *x* in the electronic supplementary material, figure D.1*j*, and display no systematic trend.

Consistent with this result, when we plotted log[*I*_G_(*x*, *t*)/*I*_G_(*x* = 0, *t*)] or log[*I*_R_(*x*, *t*)/*I*_R_(*x* = 0, *t*)] as functions of *x* for each *t*, all the data again collapsed onto a single straight line (electronic supplementary material, figure D.1*d*,*e*), implying that both distributions are of the form exp(*−κx*). The spatial decay constants *κ*_R,G_ were constant in time and did not differ significantly for *I*_G_ (*κ*_G_ = 0.66 ± 0.02 µm*^−^*^1^) and *I*_R_ (*κ*_R_ = 0.63 ± 0.02 µm*^−^*^1^) (*p* = 0.1) (figure 2*c*; electronic supplementary material, D.1*f*). This result justifies the combination of equations (2.4) and (2.5) to obtain *ω*_+_ = 0.024 ± 0.002 s*^−^*^1^ and *ω*_−_ = 0.035 ± 0.003 s*^−^*^1^ (dashed-dotted line, figures 2*e* and 8*a*).

Qualitatively similar results were obtained in all cases examined (7/7 cells).

#### 3.2.3. Cytoplasmic activation in B16-F1

**Figure 3.**
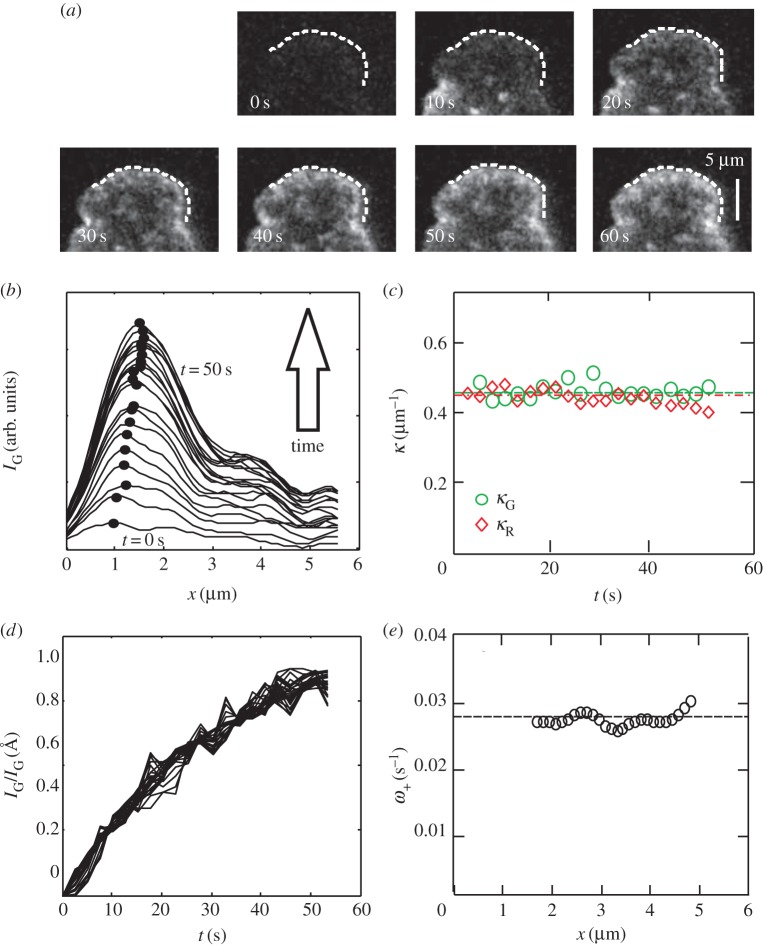
Actin assembly rate in the lamellipodium. (*a*) Cytoplasmic activation at time *t* = 0 in a B16-F1 lamellipodium. The position of the leading-edge membrane derived from the red fluorescence channel is indicated by the white dashed line. (*b*) The PA-GFP intensity *I*_G_(*x*, *t*) is plotted as a function of *x* in time steps of 2.5 s. The peak intensity *x*_max_ moves at velocity *v* ∼ 0.0061 µm s^−1^. (*c*) The spatial decay constant *κ*_G_ of each curve in (*a*) for *x* > *x*_max_ (circles, average value 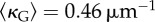, *n* = 20 points, dashed line), and the spatial decay constant *κ*_R_ derived from the mRFP profile (diamonds, average value 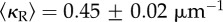, dotted-dashed line). (*d*) The PA-GFP data normalized to the fluorescence intensity at *t* → *∞* and plotted as a function of time for each *x* > *x*_max_ collapse onto master curve of the form *y* = 1 − *a* exp(*−bt*). (*e*) The temporal decay constant obtained by fitting the curves in (*b*) and associated with *ω*_+_ (see §3.2.3) was not dependent on *x* (average value 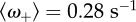, *n* = 28 points). (Online version in colour.)

The above analysis, in both HL60 cells and B16-F1 cells, yielded *ω*_+_ values that were nonzero and of comparable magnitude to *ω*_−_, suggesting that actin incorporation occurred significantly throughout the lamellipodium, and not only at the tip of the leading edge. These results are in direct contrast to recent photoactivation studies [14] but in agreement with data from speckle microscopy experiments [15,27]. To confirm our results using an independent method, we photoactivated free actin monomers in the B16-F1 cell body and monitored their gradual incorporation into the lamellipodium. In these conditions, *I*_G_(*x*, *t*) reports directly on F-actin association. Figure 3*a* shows a gradual increase in lamellipodium fluorescence after photoactivation of a large region situated outside of the imaging frame.

In our experiments, after photoactivation in the cell body, the PA-GFP intensity *I*_G_(*x*, *t*) initially increased most rapidly at the leading-edge membrane, consistent with its known high polymerization rate. However, *I*_G_(*x*, *t*) also increased simultaneously for all locations behind the leading edge membrane, albeit at a slower rate (figure 3*b*, *x* > 2 µm). A purely convective fluorescence recovery would imply that the onset of increase in PA-GFP intensity at a given *x* should be delayed by *δt* = *x*/*v*, the time necessary for the actin incorporation front to travel from the leading edge membrane. Thus, for *x* = 1 µm behind the leading edge membrane, we would expect a delay *δt* ∼ 0.9 min, easily measurable in our experimental conditions. Yet, no such delays were apparent.

When normalized to their values at long time scales *I*_G_(*x*, *t* → *∞*), the curves at each location *x* again collapsed onto one master curve (figure 3*d*; see also the electronic supplementary material, figure D.2*f*), indicating that the relative association rate *∂*log*I*_G_/*∂t* was again location-independent. These data agree with the prediction *I*_G_(*x*, *t*) = *I*_∞_(*x*)(1 − e*^−ω^* ^+^ *^t^*) (derived in the electronic supplementary material, §A.3), for a constant and *x*-independent *ω*_+_ = 0.028 ± 0.001 s*^−^*^1^, consistent with the results of figure 2. Further, the spatial decay constant *κ*_G_ of the *I*_G_ profile was also constant over time (*κ*_G_ = 0.46 ± 0.02 µm*^−^*^1^, figure 3*c*) and equal to *κ*_R_ = 0.45 ± 0.02 µm*^−^*^1^ (*p* = 0.1), obtained from the *I*_R_ data (electronic supplementary material, figure D.2). Qualitatively similar results were obtained for all cells examined (5/5 cells). These results confirm that (i) *ω*_+_ and *ω*_−_ are location- and time-independent in B16-F1 cells and (ii) actin polymerization occurs behind the leading edge membrane with an absolute rate that is proportional to the local F-actin density.

### 3.3. Filament pointed-end and Arp2/3 complex distributions mirror the F-actin distribution in the lamellipodium

The independence of the association and dissociation rates *ω*_+_ and *ω*_−_ on both *x* and *t* observed in both cell types suggested that the combination of biochemical processes that regulate actin turnover are also independent of time and location. Indeed, for *ω*_+_ and *ω*_−_ to be constant, the overall balance of actin filament homeostasis mechanisms must be the same at all locations away from the leading edge. Assuming that association and dissociation obey first-order kinetics, one would therefore expect the concentrations of actin monomer association and dissociation sites (barbed and pointed ends) to be proportional to the local F-actin density. A reasonable further hypothesis is that all of the structural and kinetic parameters influencing actin turnover dynamics are also proportional to the local F-actin density. Thus, although the absolute F-actin density varies with distance away from the leading edge membrane, the local concentrations of barbed ends, pointed ends, capped ends and filament branch points should all vary in proportion to one another. To test this hypothesis in B16-F1 cells, we compared the steady-state densities of F-actin revealed by phalloidin labelling with those of free pointed ends imaged by binding of recombinant GFP-tropomodulin and of branch points revealed by localization of the Arp2/3 complex.

**Figure 4.**
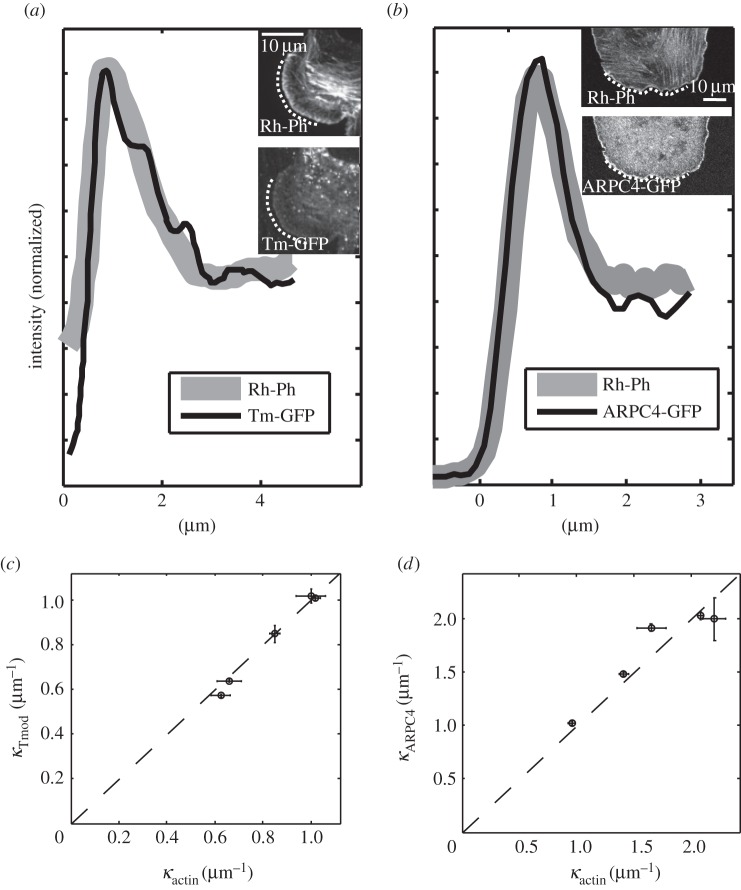
Spatial localization of pointed ends and branch points. Comparison of F-actin density distribution obtained by rhodamine-phalloidin staining (Rh-Ph, thick grey lines) with the free pointed-end distribution (tropomodulin (Tm)-GFP in *a*), and the Arp2/3 complex density distribution (ARPC4-GFP in *b*). Insets show the contour segments along which the distributions were measured. (*c*,*d*) The spatial decay constants *κ* (*x* > *x*_max_) for both Tm and ARPC4 show good consistency with the corresponding actin *κ* values (*n* = 5, each case).

To within experimental error, the spatial profile of pointed-end density across the lamellipodium closely matched the F-actin density (figure 4*a*, 5/5 lamellipodia examined). The similarity in density profiles indicates that, in the steady state, the distribution of free pointed ends (i.e. actin dissociation sites) decays exponentially as a function of *x* with the same decay constant *κ* as the F-actin density distribution, consistent with our hypothesis (figure 4*c*). Similarly, the spatial profile of branch-point density revealed by the localization of the ARPC4 subunit of the Arp2/3 complex mirrored the F-actin density profile across the lamellipodium (figure 4*b*,*d*, 5/5 lamellipodia examined). These data indicate that, in the steady state, free pointed ends and branch points are distributed throughout the lamellipodium rather than being confined to any specific region. The density of both free pointed ends and branch points decayed exponentially with distance from the leading-edge membrane with the same characteristic length scale as the F-actin density.

**Figure 5.**
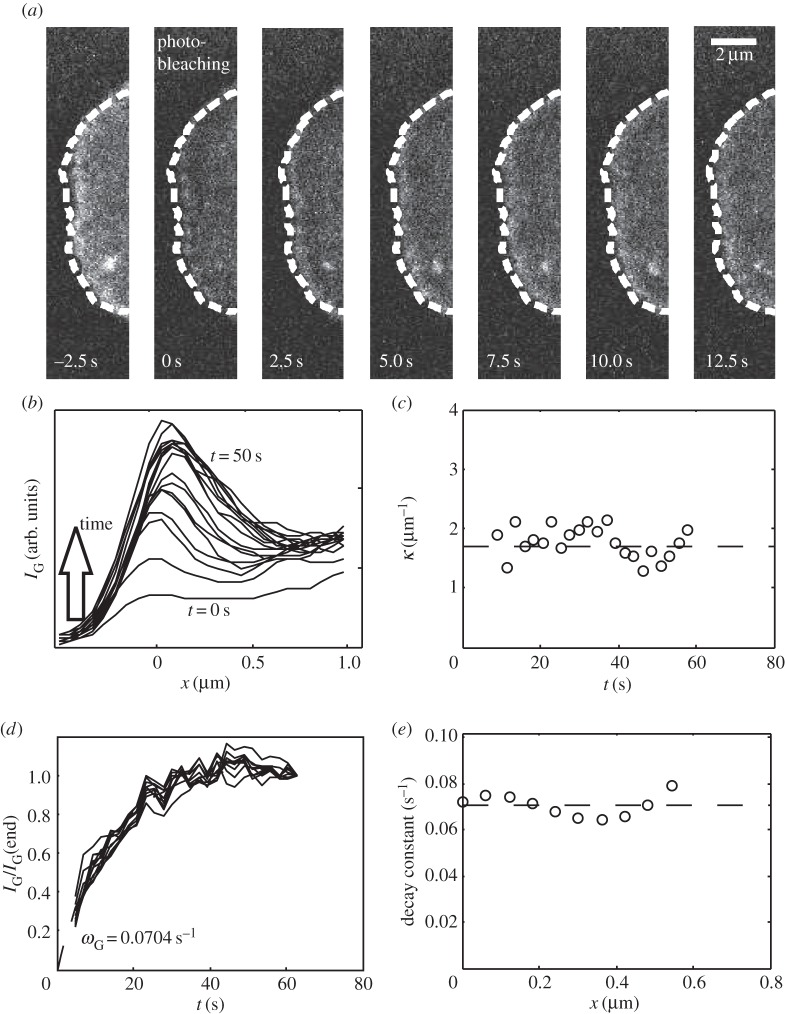
Arp2/3 turnover in the lamellipodium of B16-F1 cells. (*a*) Fluorescence recovery after photobleaching of the ARPC4-GFP subunit of the Arp2/3 complex in a B16-F1 lamellipodium. Photobleaching was effected at *t* = 0 s. (*b*) Spatial profile of ARPC4-GFP fluorescence intensity averaged over the contour shown in (*a*) and plotted at intervals of 2.5 s. (*c*) The spatial decay constant *κ*_ARPC4_, obtained from an exponential fit of each curve for all positions *x* > *x*_max_ shows no noticeable time dependence (mean value *κ*_ARPC4_ = 1.7 ± 0.1 µm*^−^*^1^, *n* = 22 points). (*d*) Intensity data shown in (*b*), normalized to values *t* → *∞* and plotted as a function of time for each position *x* > *x*_max_. All curves collapse onto a single master curve of the form *y* = *a* (1 − e*^−*ω*t^*). (*e*) Temporal decay constants *ω*, obtained by fitting each curve in (*d*). Decay constants show no noticeable *x* dependence (mean value 0.070 ± 0.002 s^−1^, *n* = 10 points).

We next investigated whether the Arp2/3 spatial density profile resulted solely from incorporation into the network at the leading edge membrane before propagating by retrograde flow (as proposed by Lai *et al*. [14]), or also from association/dissociation throughout the leading edge. Speckle microscopy experiments have highlighted the preferential incorporation of the Arp2/3 complex near the leading-edge membrane, consistent with our model, which requires the existence of a localized source term (see the electronic supplementary material, equation (B.1)) [16]. However, our focus is on dynamics in the region *behind* the leading edge membrane. To explore this region more specifically, we carried out fluorescence recovery after photobleaching (FRAP) experiments on the ARPC4 subunit of the Arp2/3 complex (figure 5*a*), thereby harvesting more Arp2/3 incorporation signals in our region of interest. Following photobleaching, the ARPC4-GFP fluorescence intensity *I*_G,ARPC4_(*x*, *t*) recovered simultaneously throughout the lamellipodium and not exclusively at the leading edge (figure 5*b*). A similar analysis as for cytoplasmic activation of actin (figure 3; electronic supplementary material, figure D.2) showed that the ARPC4-GFP fluorescence recovered exponentially. When we normalized recovery to fluorescence intensity at *t* → *∞*, all curves collapsed onto one master curve independent of their distance *x* to the leading edge membrane (figure 5*d*). This indicated that Arp2/3 complex incorporation occurred throughout the lamellipodium with a uniform effective rate constant (figure 5*e*). Furthermore, the spatial distribution of Arp2/3 complex fluorescence intensity at each time point was exponential and its spatial decay constant *κ*_ARPC4_ was constant over time (figure 5*c*). If fluorescence recovery was ‘convective’ rather than ‘reactive’, the intensity recovery within the lamellipodium would be delayed by a time proportional to distance from the leading edge. Our experimental results show no such delay (figure 5*b*,*d*). Taken together, these results suggest that Arp2/3 incorporation occurs throughout the leading edge at a constant rate and with an amplitude proportional to the local Arp2/3 density, and hence proportional also to the local F-actin density.

## 4. Theoretical model

**Figure 6.**
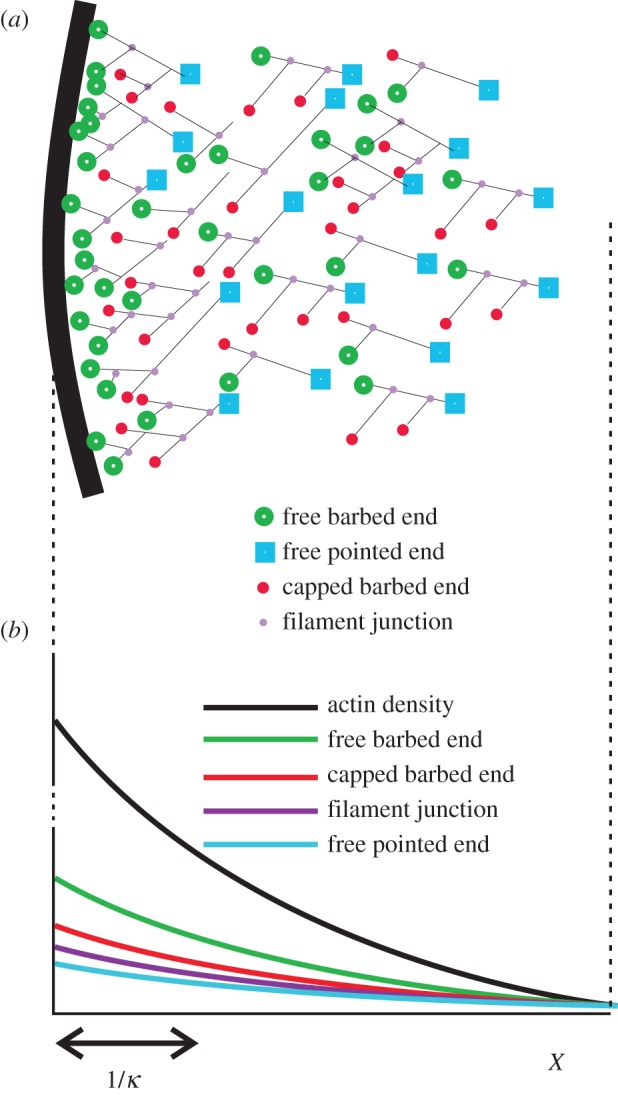
Conceptual framework for a continuum approximation of the lamellipodium. (*a*) A common representation of the lamellipodium actin system, explicitly showing filament barbed-ends, pointed-ends, and junctions. (*b*) A one-dimensional equivalent continuum representation, expressed through the concentration distribution of the filament ends. These distributions can be obtained by averaging their two-dimensional representation over the whole width of the lamellipodium. The total F-actin distribution (upper curve) is orders of magnitude greater than the other distributions, which account only for the filament ends. However, our experimental results suggest all concentrations decay exponentially with distance from the leading edge membrane with the same spatial decay constant *κ* as the F-actin concentration. (Online version in colour.)

Together, our experimental results showed that actin turnover dynamics depended only on the local F-actin network density, with no explicit dependence on distance from the leading edge membrane. The data suggest that the processes regulating the turnover of F-actin, barbed-ends, pointed-ends and branch-points are all governed by kinetic parameters that are spatially uniform behind the leading edge membrane. To explore this hypothesis theoretically and to derive testable hypotheses, we developed a mathematical description of the lamellipodium that naturally expresses these observations. Extending the approach of Mogilner *et al*. [35] and Dawes *et al*. [36], we treated the system as a continuum controlled by a set of basic biochemical processes. By modelling these generic phenomenological processes probabilistically in terms of the continuum density, rather than by starting from explicit molecular mechanisms, we minimized the assumptions on the actin system microstructure and its detailed proteic composition. This approach does not deny the complexity of lamellipodial dynamics but exploits the idea that, to a good approximation, this complexity can be treated implicitly. In numerical simulations, detailed molecular mechanisms underlying turnover of actin filaments are commonly treated explicitly (e.g. using a picture similar to figure 6*a*); this often requires assumptions on the network structure that are beyond the reach of measurement. We here used a more abstract one-dimensional representation in terms of the local actin density *A*(*x*). As suggested in the above experimental results, the main dynamic features of the system are in proportion to *A*(*x*) and our model therefore aims to clarify the consequences of this empirical property on the balance and interdependence of the processes that regulate steady-state dynamics. Our model also predicts, at least qualitatively, experimentally verifiable effects, in particular on the lamellipodium length scale, of altering basic processes such as filament capping, severing and side branching by genetic or pharmacological intervention in steady-state conditions.

### 4.1. Derivation

We here provide an outline of the model derivation; a more thorough derivation is given in the electronic supplementary material, B. The main model assumption is that the rates of actin incorporation and dissociation are proportional to the local concentrations of filament barbed and pointed ends, *c*_B_(*x*, *t*) and *c*_P_(*x*, *t*). Thus, following equations (2.1)–(2.2), the actin association and dissociation rates in the network rest frame are written, respectively, *ω*_+_*A*(*x*, *t*) = *k*_+_*c*_B_(*x*, *t*) and *ω*_−_*A*(*x*, *t*) = *k*_−_*c*_P_(*x*, *t*), where *k_±_* are constant rates of actin incorporation and dissociation at filament ends. Other biochemical processes are assumed to contribute to the overall dynamics only via their effect on *c*_B_ and *c*_P_. The processes used in the model and their impact on these distributions are listed in table 1. Filament severing (at rate *k*_S_) locally creates one barbed and one pointed end. Barbed-end capping (*k*_C_) removes one barbed end and augments the concentration *c*_C_(*x*, *t*) of capped barbed ends. The creation of a filament branch point along an existing filament (*k*_B_) generates a free barbed end and an inactive pointed end (*c*_J_(*x*, *t*)), whereas an unbranching event (*k*_U_) transforms an inactive pointed end into an active one. We assume that a filament disintegrates completely when a pointed end catches up with a capped barbed end, an event that occurs at an estimated frequency *k*_−_*c*_C_*c*_P_/*A*. This is a reasonable first approximation. In a continuum model such as ours, the individual identity of filaments and their ends is neglected in favour of the mean-field approach, so proximity of plus and minus ends does not always imply that a single filament has disassembled. Our approximation is exact on the assumption that the probability that a given free pointed end is adjacent to a capped barbed end on the same filament equals the local mean-field capped-end probability, *c*_C_/*A*. The distributions thus obey the differential equations
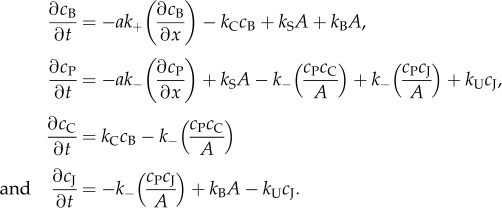
The terms with *∂*/*∂x* represent the advancement of each barbed/pointed end by a distance *a* (∼ half a monomer size). Barbed ends all advance at the speed4.1
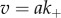
and pointed ends at a different speed *ak*_−_, both in the direction of cell motion. The solution to each distribution *A*, *c*_B_, *c*_P_, *c*_C_ and *c*_J_ (see the electronic supplementary material, B) naturally assumes the form ∼exp(*κx*) with4.2
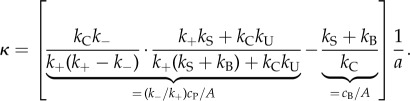
This equation provides a simple handle for quantifying changes in the lamellipodium length scale, a directly measurable parameter, in terms of generic biochemical processes, modifiable experimentally by pharmacological or genetic intervention (table 2).

**Table 1.** Kinetic formulation of network regulatory processes used in the theoretical model, expressed in terms of concentrations of F-actin (*A*), free barbed ends (*c*_B_), free pointed ends (*c*_P_), capped barbed ends (*c*_C_) and capped pointed ends (*c*_J_). A ‘+’ represents the generation of a new filament end, and ‘−’ its removal by a given process.

event	*c*_B_	*c*_P_	*c*_C_	*c*_J_	kinetic expression
severing	+	+			*k*_S_*A*
side branching	+			+	*k*_B_*A*
pointed meets barbed end		−	−		*k*_−_*c*_C_*c*_P_/*A*
barbed-end capping	−		+		*k*_C_*c*_B_
pointed-end uncapping		+		−	*k*_U_*c*_J_
pointed end meets filament junction		+		−	*k*_−_*c*_P_*c*_J_/*A*

**Table 2.** Predicted qualitative changes (see equation (4.2) and in electronic supplementary material, equations (B.6)–(B.10) in the concentrations of barbed-ends (*c*_B_), pointed-ends (*c*_P_), capped barbed-ends (*c*_C_), and capped pointed-ends (*c*_J_), and of the spatial decay constant *κ* for single changes in biochemical reaction rates (polymerization *k*_+_, depolymerization *k*_−_, severing *k*_S_, capping *k*_C_, and branching *k*_B_). ‘
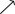
’ indicates an increase, ‘
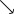
’ a decrease, ‘→’ no dependence, and double arrows the existence of several condition-dependent regimes.

parameter	symbol	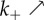	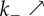	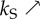	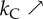	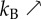
barbed ends	*c*_B_	→	→	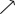	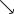	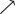
pointed ends	*c*_P_	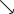	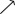	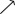 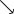	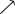	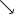
capped barbed ends	*c*_C_	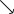	→	→	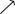	→
capped pointed ends	*c*_J_	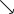	→	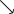	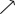	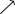 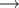
spatial decay constant	*κ*	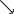	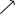	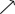 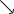	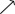	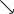

#### 4.1.1. Dependence of the lamellipodium width on side-branching rate *k*_B_

**Figure 7.**
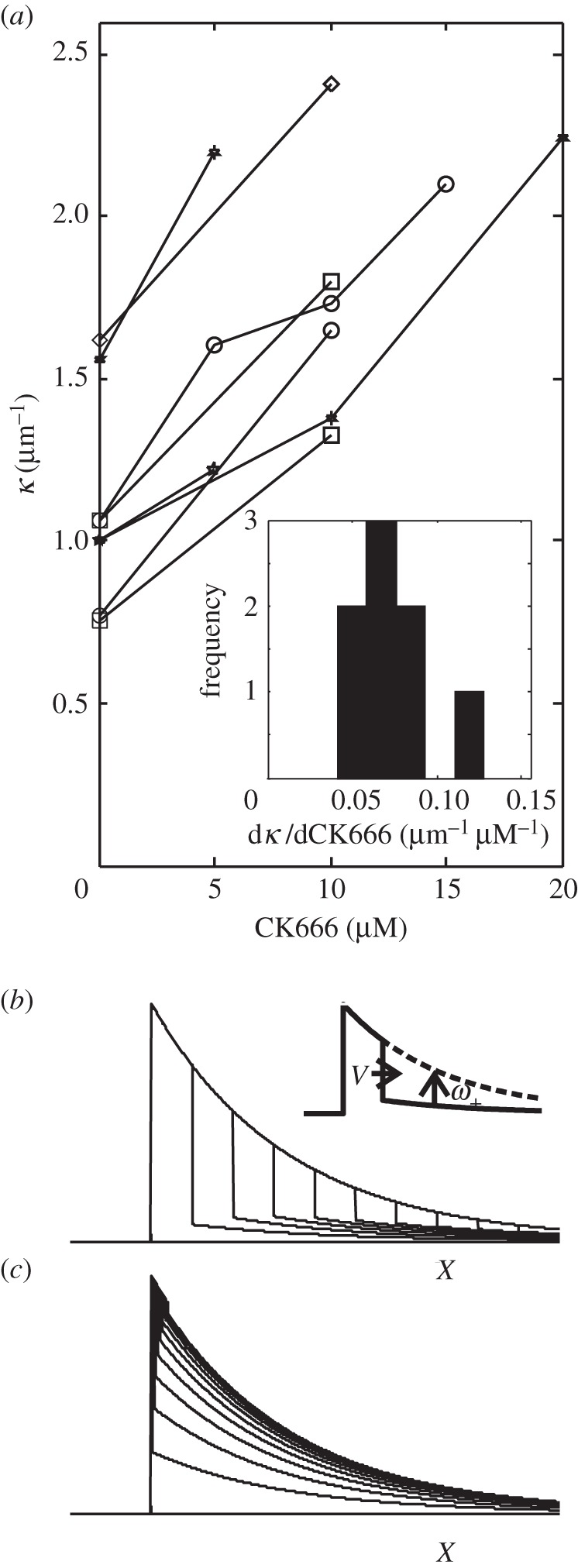
Experimental model predictions and experimental verification in B16-F1 cells. (*a*) Response of the spatial decay constant *κ* to subcritical doses of Arp2/3 complex inhibitor CK666 (*N* = 8 cells examined). Gradients for each cell are shown in the inset histogram. (*b*,*c*) Qualitative prediction of the fluorescence profile recoveries in hypothetical FRAP experiments in the limits (*b*) 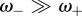 and (*c*) 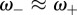. The inset schematic in (*b*) identifies the two sources of recovery: the recovery front from the leading edge advances rearward at speed *v* = (*ω*_−_ − *ω*_+_)/*κ*, whereas recovery throughout the lamellipodium occurs exponentially with time constant *ω*_+_.

Equation (4.2) predicts an approximately linear increase in the spatial decay constant *κ* with decreasing branching rate *k*_B_, at least in regimes where filament severing dominates branch formation (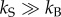). To test this prediction, we applied increasing sub-critical doses of the Arp2/3 inhibitor CK666 in 5 µM increments [37]. After each increment in CK666 concentration, we waited several minutes for cells to reach a new steady state and measured the new spatial decay constant *κ* from the intensity distribution in actin-mRFP. This yielded an experimental curve relating *κ* to CK666 concentration and hence decreasing branching rate *k*_B_ (figure 7, *N* = 8 cells). No stable lamellipodia were observed for doses exceeding 20 µM CK666. Consistent with our theoretical predictions, our experimental results revealed a progressive linear increase in *κ* with increasing dose of CK666. The last term of equation (4.2) suggests that this increase in *κ* reflects the balance between the branching and capping rates (*k*_B_/*k*_C_), which directly reflects the relative concentration of barbed ends (*c*_B_/*A*). Whereas side branching creates new free barbed ends, capping removes free barbed ends, but neither process directly alters the free depolymerizing pointed-end density. CK666 thus diminishes the relative barbed-end density, shifting the dynamic balance towards more depolymerization.

## 5. Discussion

We have examined commonalities in the steady-state lamellipodial actin association and dissociation kinetics in motile HL60 cells and non-motile B16-F1 cells. Both systems displayed first-order kinetics throughout the lamellipodium, described by rate constants that were independent of distance from the leading edge. As a consequence, the actin density distribution decays exponentially with distance from the leading edge membrane. To within measurement error, the branch-point and free pointed-end distributions also decayed exponentially with distance with the same decay constant *κ*, suggesting that the length scale 1/*κ* represents an emergent property of the lamellipodium. These results led us to hypothesize that the overall kinetics of biochemical reactions in the network were also effectively uniform across the lamellipodium. We therefore developed a simple model to relate these observations to the broad classes of known actin turnover mechanisms, and showed that the length scale *L* = 1/*κ* can be used as a simple handle to characterize the effect of perturbations.

The location of actin incorporation, either at the front membrane [14,24–26] or over a more extended region within the leading edge [15,27], has been subject to debate. Our results unambiguously show incorporation by both modes simultaneously, as evidenced by the finite *ω*_+_ and the constant network flow emanating from the front. Earlier studies, supporting one mode or the other, are therefore not necessarily contradictory, as the choice of observation method could emphasize one mode over the other. Our theoretical model sought to reconcile the apparent contradiction in terms of the balance of association and dissociation rates. Following photobleaching, rapid polymerization at the B16-F1 leading edge generates a fluorescent front that sweeps through the entire lamellipodium over a time scale *τ*_front_ ∼ *L*/*v* = 1/(*ω*_−_ − *ω*_+_) (‘convective recovery’—equation (2.4)). Meanwhile, polymerization elsewhere in the lamellipodium enables fluorescence to recover its steady-state distribution on a time scale 1/*ω*_+_ in the regions not yet reached by the recovery front (‘reactive recovery’—equation (2.5)). Such reactive recovery may go unnoticed if it is much slower than convective recovery, i.e. when local dissociation is much faster than association (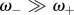). If, instead, *ω*_−_ and *ω*_+_ have similar magnitudes, steady-state fluorescence recovers faster reactively than convectively. Our numerical framework could replicate both behaviours: convective recovery from the front (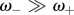) or recovery throughout the lamellipodium for 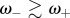. (In both cases, *ω*_−_ > *ω*_+_ is a *sine qua non* condition for the steady state—equation (2.4).) Interestingly, both convective and reactive recoveries have also been documented for Arp2/3 complex components [14,16] and in each case, the type of Arp2/3 recovery matched the type of actin recovery. Based on the known role of the Arp2/3 complex in actin polymerization in the lamellipodium, this suggests that differences in spatial regulation of Arp2/3 complex activation/inactivation may be the origin of the observed differences in F-actin turnover kinetics: slower inactivation or less stringent spatial regulation of WAVE family proteins may lead to nucleation over a larger spatial domain. In summary, we argue that reactive actin association occurs throughout the lamellipodium but that differences in the relative magnitudes of the rates of actin association and dissociation may lead to this effect being obscured in some experiments.

**Figure 8.**
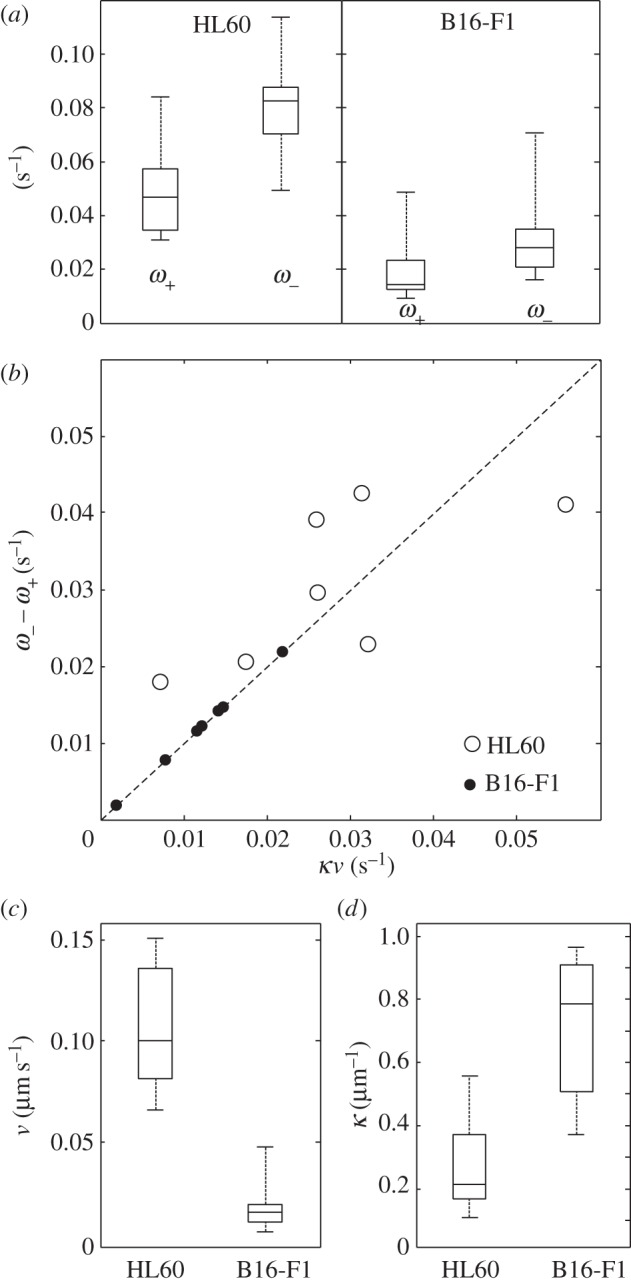
Kinetic parameters and reaction rates in the lamellipodium of B16-F1 and HL60 cells. (*a*) Box-and-whisker plots for *ω*_+_ and *ω*_−_ measured in the HL60 and B16-F1 cells. Average values: 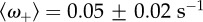, 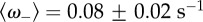 for HL60 (*n* = 7); 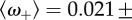 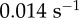, 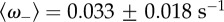 for B16-F1 (*n* = 6). The central line in the boxes represents the median; the box edges are the 25th and 75th percentiles; the whiskers cover the full range of measured values. 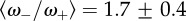 for the HL60 cells and 1.7 ± 0.5 for the B16-F1. (*b*) The rate difference *ω*_−_ − *ω*_+_ plotted as a function of *κv* for the HL60 (open symbols) and B16-F1 cells (closed symbols). The points are predicted by equation (2.4) to lie on the line of unit gradient (dashed line). Box-and-whisker plots comparing (*c*) the network flow velocity *v* (mean ± s.d., HL60: 0.10 ± 0.03 µm s^−1^; B16-F1: 0.018 ± 0.014 µm s^−1^) and (*d*) the spatial decay constant *κ* (HL60: 0.27 ± 0.15 µm*^−^*^1^; B16-F1: 0.71 µm*^−^*^1^) for both cell types.

The coexisting contributions of convective and reactive fluorescence recoveries in a steady-state lamellipodium provide complementary insight into the dynamics of the underlying actin system. Convective recovery essentially expresses the rate of monomer incorporation at barbed ends at the tip of the leading edge, associated with the rearward flow velocity *v*; whereas the reactive mode reflects the combined effects of many additional processes that are assimilated phenomenologically into the association and dissociation rate constants, *ω*_+_ and *ω*_−_. These parameters describe the punctual mechanistic behaviour of the network, and they in turn determine the more global geometrical features of the whole lamellipodium, such as the spatial decay constant *κ*. This in turn may impact physiological function. The steady-state constraint *κv* = *ω*_−_ − *ω*_+_ (equation (2.4)) holds for both the HL60 and B16-F1 cells because of the scaling properties observed in both cell types (figure 8*b*). Figure 8 shows that *ω*_+_ and *ω*_−_ in the HL60 cells are both greater by a factor of approximately 2.4 compared to the B16-F1 cells. The ratio *ω*_−_/*ω*_+_ ≈ 1.7, however, is remarkably similar. The velocities *v* differ even more significantly by a factor of almost 6. Consistent with equation (2.4), *κ* shows the opposite trend, being greater in the B16-F1 cells by a factor of 2.6. This analysis therefore suggests that the greater width of the HL60 lamellipodium (smaller *κ*) results from the greater value of *v*, which dominates the more modest increase in *ω*_−_ − *ω*_+_.

Theoretical analysis and simulations are essential complements to experiments for quantitatively understanding lamellipodium dynamics. Overwhelmingly, existing models tend to use a ‘bottom-up’ approach: starting from a combination of specific biochemical processes and a set of best parameter estimates, they seek to reproduce experimental observations. Inevitably, any theoretical model relies on simplifying assumptions, either with regard to the processes considered, the values of key parameters, or the topology of the F-actin network. The validity of a simulation therefore hinges on whether all relevant processes are taken into account. This is a challenging task, as the full list of relevant proteins and their precise *in vivo* behaviour remain uncertain. To avoid this problem, we considered the system from a more abstract perspective, and formally reduced its dynamics to that of actin association and dissociation sites, without involving the protein players explicitly. This ‘top-down’ approach sought to minimize assumptions on the detailed network structure or biochemical processes involved. Prompted by the empirical scaling laws we report, we considered the system as a single-phase continuum. We developed a theoretical framework for treating the consequences of network structure and generic biochemical processes from a purely probabilistic perspective without implementing them explicitly at a molecular level, thereby avoiding making many assumptions. Our underlying assumption was that all the biochemical processes (capping, severing, side branching, etc.) contributed to turnover dynamics only by affecting the distributions of barbed and pointed filaments ends, *c*_B_ and *c*_P_. The corresponding kinetic equations yielded analytical solutions that naturally reproduced the empirical scaling law. Another outcome of the solution is that the characteristic length scale *L* = 1/*κ* of the lamellipodium emerges as an experimental observable that can be reliably estimated through a variety of experimental techniques. Our minimal model can thus potentially unify a wide body of observations on the lamellipodium of motile and immotile cells based on the class of biochemical process each protein contributes to and the nature of the chemical or genetic perturbation.

Equation (4.2) relates the directly measurable length scale *L* with basic underlying phenomenological processes in the steady state. Because both the mobile HL60 and immobile B16-F1 cells displayed the same scaling behaviour, this equation applies to both cell types. Our model predicts that *L* decreases with decreasing side-branching rate *k*_B_, as observed in our experiments after perturbation by CK666, as well as in experiments overexpressing tropomyosin to prevent Arp2/3 complex attachment to the side of existing filaments [38]. Also consistent with this observation, increases in *k*_B_ resulting from tropomyosin depletion produce broader lamellipodia [39]. Equation (4.2) also predicts an increase in *L* by a decrease in the spontaneous unbranching rate *k*_U_. Interestingly, perturbations to ATP hydrolysis in Arp2/3 complex subunits broaden lamellipodia [40]. The observed increased longevity of these Arp2/3 mutants in the F-actin network can, within our formalism, be associated with a decrease in *k*_U_.

Many experiments have examined the effects of ADF/cofilin on the lamellipodium, revealing a myriad of sometimes contradictory effects [23,39,41]. Our model may provide insight into this paradox. Experimental evidence points to a complex interaction between cofilin and the actin network, involving either filament severing or end depolymerization activities. Within the model, cofilin-mediated pointed-end depolymerization amounts primarily to an increase of *k*_−_. This parameter appears only in the first term of equation (4.2). Equation (4.2) would therefore predict a decrease in the lamellipodium turnover time (*L*/*v*). On the other hand, severing activity is expressed by *k*_S_ and therefore in principle affects both terms, via *c*_P_ and *c*_B_, through the simultaneous creation of both pointed and barbed ends. Either reduction or increase in the turnover time may then result in different experimental systems that operate in distinct regions of the phase diagram of cofilin activity.

Experimentally, decreases in cofilin activity obtained through siRNA-mediated depletion of cofilin or depletion of the cofilin phosphatase slingshot [39] both lead to broader lamellipodia, as expected from equation (4.2) if these perturbations principally decrease the depolymerization rate *k*_−_ rather than *k*_S_. Decreases in severing activity alone associated with depletion of twinfillin led to broader lamellipodia [39]. By contrast, some experiments have reported increases in lamellipodium width associated with increased cofilin activity [23]. In these, exogenous caged cofilin was microinjected into cells and suddenly uncaged by exposure to UV light. These seemingly contradictory effects may be explained by the balance between the relative significance of the two terms in equation (4.2). This balance is controlled by the magnitude of *k*_C_ in particular. A large value of *k*_C_ suppresses barbed ends and decreases *c*_B_, making the second term in equation (4.2) less significant. However, a large *k*_C_ also favours larger *c*_P_ (electronic supplementary material, equation (B.7)) and therefore enhances the effect of *k*_−_ in the first term of equation (4.2). Depletion of capping protein led to loss of lamellipodia [39], perhaps because the suppression of *k*_C_ dramatically enhanced the second term and tended to produce an effectively negative *κ*, making the lamellipodium unviable. Equivalently, the increase in *c*_B_ was such that the steady-state condition *ω*_−_ > *ω*_+_ was no longer satisfied.

Other experiments examining the role of cofilin inactivation by Lim-kinase revealed a decrease in retrograde flow velocity *v* [41], associated with overexpression of Lim-kinase, and an increase for Lim-kinase depletion. This behaviour can be understood within our model if cofilin activity also affects *v* via *k*_+_ (equation (4.1)), possibly by generating more cytoplasmic G-actin monomers [42].

In summary, our kinetic model provides a simple framework for considering the effect of perturbations to proteins that play a role in actin dynamics through the measurement of the lamellipodium characteristic length scale *L* = 1/*κ* and retrograde flow *v*, simple observables that can be quantified with minimal technical difficulty. In consequence, we anticipate that our study will pave the way for a systematic dissection of the role and importance of candidate proteins in lamellipodial dynamics.

## Funding statement

A.L. and K.W. were supported by a BBSRC project grant (BB/F021402) to G.C. and T.D. G.C. was supported by a University Research Fellowship from the Royal Society. M.F. was funded by a Human Frontier Science Program Young Investigator grant to G.C. (grant RGY0067/2008). R.T. was partly supported by BBSRC project grants BB/F021402 and BB/F019769 to G.C. The authors wish to acknowledge funding from the UCL Comprehensive Biomedical Research Centre for funding of microscopy equipment.

## References

[1] Peskin C Odell G Oster G 1993 Cellular motions and thermal fluctuations: the Brownian ratchet. Biophys. J. 65, 316–324. (10.1016/S0006-3495(93)81035-X) 8369439 PMC1225726

[2] Mogilner A Oster G 1996 Cell motility driven by actin polymerization. Biophys. J. 71, 3030–3045. (10.1016/S0006-3495(96)79496-1) 8968574 PMC1233792

[3] Giannone G 2007 Lamellipodial actin mechanically links myosin activity with adhesion-site formation. Cell 128, 561–575. (10.1016/j.cell.2006.12.039) 17289574 PMC5219974

[4] Prass M Jacobson K Mogilner A 2006 Direct measurement of the lamellipodial protrusive force in a migrating cell. J. Cell Biol. 174, 767–772. (10.1083/jcb.200601159) 16966418 PMC2064331

[5] Dogterom M Janson M Faivre-Moskalenko C van der Horst A Kerssemakers JWJ Tanase C Mulder BM 2002 Force generation by polymerizing microtubules. Appl. Phys. A 75, 331–336. (10.1007/s003390201342)

[6] Marcy Y Prost J Carlier MF Sykes C 2004 Forces generated during actin-based propulsion: a direct measurement by micromanipulation. Proc. Natl Acad. Sci. USA 101, 5992–5997. (10.1073/pnas.0307704101) 15079054 PMC395911

[7] Mitchison T Kirschner M 1988 Cytoskeletal dynamics and nerve growth. Neuron 1, 761–772. (10.1016/0896-6273(88)90124-9) 3078414

[8] Aratyn-Schaus Y 2008 Clutch dynamics. Science 322, 1646–1647. (10.1126/science.1168102) 19074337

[9] Fournier M Sauser R Ambrosi D Meister JJ Verkhovsky AB 2010 Force transmission in migrating cells. J. Cell Biol. 188, 287–297. (10.1083/jcb.200906139) 20100912 PMC2812525

[10] Svitkina TM Verkhovsky AB McQuade KM Borisy GG 1997 Analysis of the actin–myosin II system in fish epidermal keratocytes: mechanism of cell body translocation. J. Cell Biol. 139, 397–415. (10.1083/jcb.139.2.397) 9334344 PMC2139803

[11] Dyche Mullins R Heuser J Pollard T 1998 The interaction of Arp2/3 complex with actin: nucleation, high affinity pointed end capping, and formation of branching networks of filaments. Proc. Natl Acad. Sci. USA 95, 6181–6186. (10.1073/pnas.95.11.6181) 9600938 PMC27619

[12] Pollard T 1986 Rate constants for the reactions of ATP- and ADP-actin with the ends of actin filaments. J. Cell Biol. 103, 2747–2754. (10.1083/jcb.103.6.2747) 3793756 PMC2114620

[13] Pollard T Borisy GG 2003 Cellular motility driven by assembly and disassembly of actin filaments. Cell 112, 453–465. (10.1016/S0092-8674(03)00120-X) 12600310

[14] Lai FP 2008 Arp2/3 complex interactions and actin network turnover in lamellipodia. EMBO J. 27, 982–992. (10.1038/emboj.2008.34) 18309290 PMC2265112

[15] Ponti A 2004 Two distinct actin networks drive the protrusion of migrating cells. Science 305, 1782–1786. (10.1126/science.1100533) 15375270

[16] Miyoshi T Tsuji T Higashida C 2006 Actin turnover-dependent fast dissociation of capping protein in the dendritic nucleation actin network: evidence of frequent filament severing. J. Cell Biol. 175, 947–955. (10.1083/jcb.200604176) 17178911 PMC2064704

[17] Tsuji T Miyoshi T Higashida C Narumiya S Watanabe N 2009 An order of magnitude faster AIP1-associated actin disruption than nucleation by the Arp2/3 complex in lamellipodia. PLoS ONE 4, e4921 (10.1371/journal.pone.0004921) 19290054 PMC2654150

[18] Miyoshi T Watanabe N 2013 Can filament treadmilling alone account for the F-actin turnover in lamellipodia? Cytoskeleton 70, 179–190. (10.1002/cm.21098) 23341338

[19] Campellone KG Welch MD 2010 A nucleator arms race: cellular control of actin assembly. Nat. Rev. Mol. Cell Biol. 11, 237–251. (10.1038/nrm2867) 20237478 PMC2929822

[20] Carlier MF Laurent V Santolini J Melki R Didry D Xia G-X Hong Y Chua N-H Pantaloni D 1997 Actin depolymerizing factor (ADF/cofilin) enhances the rate of filament turnover: implication in actin-based motility*.* J. Cell Biol. 136, 1307–1323. (10.1083/jcb.136.6.1307) 9087445 PMC2132522

[21] Hotulainen P 2004 Actin-depolymerizing factor and cofilin-1 play overlapping roles in promoting rapid F-actin depolymerization in mammalian nonmuscle cells. Mol. Biol. Cell 16, 649–664. (10.1091/mbc.E04-07-0555) 15548599 PMC545901

[22] Kueh HY Charras GT Mitchison TJ Brieher WM 2008 Actin disassembly by cofilin, coronin, and Aip1 occurs in bursts and is inhibited by barbed-end cappers. J. Cell Biol. 182, 341–353. (10.1083/jcb.200801027) 18663144 PMC2483518

[23] Ghosh M Song X Mouneimne G Sidani M Lawrence DS Condeelis J 2004 Cofilin promotes actin polymerization and defines the direction of cell motility. Science, 304, 743–746. (10.1126/science.1094561) 15118165

[24] Symons MH Mitchison TJ 1991 Control of actin polymerization in live and permeabilized fibroblasts. J. Cell Biol. 114, 503–513. (10.1083/jcb.114.3.503) 1860882 PMC2289085

[25] Wang YL 1985 Exchange of actin subunits at the leading edge of living fibroblasts: possible role of treadmilling. J. Cell Biol. 101, 597–602. (10.1083/jcb.101.2.597) 4040521 PMC2113673

[26] Theriot JA Mitchison TJ 1991 Actin microfilament dynamics in locomoting cells. Nature 352, 126–131. (10.1038/352126a0) 2067574

[27] Watanabe N Mitchison TJ 2002 Single-molecule speckle analysis of actin filament turnover in lamellipodia. Science 295, 1083–1086. (10.1126/science.1067470) 11834838

[28] Holmes WR Edelstein-Keshet L 2012 A comparison of computational models for eukaryotic cell shape and motility. PLoS Comput. Biol. 8, e1002793 (10.1371/journal.pcbi.1002793) 23300403 PMC3531321

[29] Carlsson AE Sept D 2008 Mathematical modeling of cell migration. Methods Cell Biol. 84, 911–937. (10.1016/S0091-679X(07)84029-5) 17964954

[30] Patterson GH 2002 A photoactivatable GFP for selective photolabeling of proteins and cells. Science 297, 1873–1877. (10.1126/science.1074952) 12228718

[31] Reymann A-C Suarez C Guérin C Martiel J-L Staiger CJ Blanchoin L Boujemaa-Paterski R 2011 Turnover of branched actin filament networks by stochastic fragmentation with ADF/cofilin. Mol. Biol. Cell 22, 2541–2550. (10.1091/mbc.E11-01-0052) 21613547 PMC3135479

[32] Watanabe N 2010 Inside view of cell locomotion through single-molecule: fast F-/G-actin cycle and G-actin regulation of polymer restoration. Proc. Jpn Acad. B Phys. Biol. Sci. 86, 62–83. (10.2183/pjab.86.62) PMC3417570 20075609

[33] Tardy Y McGrath JL Hartwig JH Dewey CF 1995 Interpreting photoactivated fluorescence microscopy measurements of steady-state actin dynamics. Biophys. J. 69, 1674–1682. (10.1016/S0006-3495(95)80085-8) 8580311 PMC1236401

[34] Heit B Kubes P 2003 Measuring chemotaxis and chemokinesis: the under-agarose cell migration assay. Sci. Signal. 2003, pl5 (10.1126/stke.2003.170.pl5) 12591998

[35] Mogilner A Edelstein-Keshet L 2002 Regulation of actin dynamics in rapidly moving cells: a quantitative analysis. Biophys. J. 83, 1237–1258. (10.1016/S0006-3495(02)73897-6) 12202352 PMC1302225

[36] Dawes AT Bard Ermentrout G Cytrynbaum E Edelstein-Keshet L 2006 Actin filament branching and protrusion velocity in a simple 1D model of a motile cell. J. Theor. Biol. 242, 265–279. (10.1016/j.jtbi.2006.02.017) 16600307

[37] Nolen B Tomasevic N Russell A Pierce D 2009 Characterization of two classes of small molecule inhibitors of Arp2/3 complex. Nature 460, 1031–1035. (10.1038/nature08231) 19648907 PMC2780427

[38] Gupton SL 2005 Cell migration without a lamellipodium: translation of actin dynamics into cell movement mediated by tropomyosin. J. Cell Biol. 168, 619–631. (10.1083/jcb.200406063) 15716379 PMC2171771

[39] Iwasa J Mullins R 2007 Spatial and temporal relationships between actin-filament nucleation, capping, and disassembly. Curr. Biol. 17, 395–406. (10.1016/j.cub.2007.02.012) 17331727 PMC3077992

[40] Ingerman E Hsiao JY Mullins R 2013 Arp2/3 complex ATP hydrolysis promotes lamellipodial actin network disassembly but is dispensable for assembly. J. Cell Biol. 200, 619–633. (10.1083/jcb.201211069) 23439681 PMC3587832

[41] Ohashi K Fujiwara S Watanabe T Kondo H Kiuchi T Sato M Mizuno K 2011 LIM kinase has a dual role in regulating lamellipodium extension by decelerating the rate of actin retrograde flow and the rate of actin polymerization. J. Biol. Chem. 286, 36 340–36 351. (10.1074/jbc.M111.259135) PMC3196069 21868383

[42] Kiuchi T Ohashi K Kurita S Mizuno K 2007 Cofilin promotes stimulus-induced lamellipodium formation by generating an abundant supply of actin monomers. J. Cell Biol. 177, 465–476. (10.1083/jcb.200610005) 17470633 PMC2064820

